# The Association between Patient Activation and Outcomes among Severely Mentally Ill Patients

**DOI:** 10.1007/s11414-020-09731-3

**Published:** 2020-11-17

**Authors:** Felicia Forma, Jennifer Clerie, Tigwa Davis, Kelly Clovie, Charles Ruetsch

**Affiliations:** 1 Otsuka Pharmaceutical Development & Commercialization, Inc, Princeton, NJ 08540 USA; 2 Health Analytics, LLC 9200 Rumsey Rd Suite 215, Columbia, MD 21045 USA

## Abstract

Little is known about the association between patient activation, health, service utilization, and cost among mental health (MH) patients. Patients aged 18 to 64 with schizophrenia (Sz, *n* = 43), bipolar disorder (BD, *n* = 59), or major depressive disorder (MDD, *n* = 34) completed the Patient Activation Measure for Mental Health (PAM-MH), the Colorado Symptom Index, demographic, socioeconomic, treatment, and social support questionnaire items. Average PAM-MH score indicated BD patients the most activated (66.6 ± 17.5), Sz (57.4 ± 10.4) less activated, and MDD the least activated (55.4 ± 14.6). The MDD cohort had the highest ($27,616 ± 26,229) and the BD had the lowest total annual healthcare cost ($18,312 ± 25,091). PAM-MH score was inversely correlated with healthcare costs and regression analysis showed a PAM-MH score × gender interaction. The strongest negative relationship between PAM and cost was for males. These analyses support the inverse association between PAM-MH and healthcare service utilization and cost.

## Introduction

Serious mental illness (SMI), including schizophrenia (Sz), bipolar disorder (BD), and major depressive disorder (MDD), affect approximately 4.5% of the adult US population.^1^ These diseases are often difficult to treat and are associated with elevated healthcare cost, which is largely driven by relapse events.^2–8^ Although only a small percentage of individuals are impacted by these disorders, healthcare costs to treat SMI patients are estimated at $148 billion annually and account for approximately 10% of total annual direct healthcare costs in the USA.^1^

As is the case for primary healthcare, treatment of mental health disorders is moving from a paternalistic model to one that places greater emphasis on patient involvement and engagement in their own personalized treatment regimens.^9,10^ In primary care, high levels of patient activation, defined as “*a patient’s willingness and ability to take independent actions to manage their health and care,*”^11^ p207 are associated with a lower cost of care likely driven by increased use of both healthy behaviors and preventative care.^11,12^ Further, increases in patient activation are associated with stepwise improvements in health outcomes including clinical indicators, medication adherence, and health-related quality of life (HRQoL).^11^

Patients with severe mental illness often face greater behavioral and personal care challenges. Nevertheless, they show interest in and actively engage their healthcare providers and collaborate in shared healthcare decision-making in both primary care as well as mental healthcare.^13,14^ Accordingly, the few studies of patient activation among SMI populations mirror studies conducted among patients with chronic physical diseases, like cardiovascular disease and diabetes. For example, increased levels of patient activation are associated with increased self-management behavior and reduced substance abuse.^15^ Similarly, participation in health-focused intervention programs increases patient activation levels and results in increased adherence, improved HRQoL, and greater use of primary care services among SMI populations.^16^ These findings suggest that increased activation among SMI patients may be associated with improved health outcomes and reduced overall cost of care. Decreases in healthcare costs in activated populations are expected to result from reduced relapse events and the associated use of high-cost venue services, possibly driven by increased medication adherence.^16,17^ Yet, the high rate of comorbidities among SMI patients provides another opportunity for improving the use of health-related behaviors. Such an improvement could result in additional cost offset through improved self-care and management of chronic medical conditions. The present study combined patient self-report survey data with administrative claims data to assess the impact of patient activation on the management of their own healthcare thereby potentially reducing utilization of high-cost services and total cost of care. The relationship between activation, treatment regimens, patient outcomes, service utilization, and costs was evaluated to better characterize the impact of patient engagement in the healthcare system not only for the treatment of mental health disorders but also on other healthcare costs incurred by an SMI population.

## Methods

The sample of study participants *N* = 136 with Sz (*n* = 43), BD (*n* = 59), or MDD (*n* = 34), derived from a large national Managed Medicaid organization , were recruited through contracted sites. Once verified that they met inclusion and exclusion criteria, they completed the survey battery.

### Inclusion criteria

- Diagnosis of schizophrenia, BD, or MDD
  - A diagnosis of Sz (ICD-9 CM 295.x/ICD-10 CM F20.x), BD (ICD-9 CM 296.0–296.1, 296.4–296.8/ICD-10 CM F31.x), or MDD (ICD-9 CM 296.2–296.3, 311/ICD-10 CM F32.x-F33.x) was determined by the presence of at least two claims with a relevant diagnostic code in the primary position within a 1-year period during the case-finding window.
  - All diagnoses were confirmed by the study site prior to recruitment.
- Insured through national managed Medicaid organization
  - Continuous eligibility for at least 1 year prior to survey administration
- Aged between 18 and 64 at the time of survey administration
- Able to read English
- Able to serve as their own legally authorized representative
  - Legal status was confirmed by the study site

### Participant and site identification

Potential study participant recruitment sites (SPRSs) were identified using administrative claims. Patients insured through a national managed Medicaid organization who have a diagnosis of schizophrenia, BD, or MDD were identified using the relevant International Classification of Diseases (ICD) codes present within claims data. Cases that met inclusion criteria were sorted and counted by the provider using the national provider identifier (NPI). Providers with the highest number of potential study participants in their patient panels were considered high-volume providers for each of the three diagnoses. From the list of potential providers, high-volume mental health practices were initially targeted as potential SPRSs.

### Data source

Study data were derived from two sources: administrative healthcare claims and participant survey responses.

### Administrative claims

The study included the integration of survey and administrative claims data. The study data partner (a national managed Medicaid organization) transferred medical and pharmacy claims to Health Analytics, LLC where they were used for both recruitment and analytic purposes. Claims data were used to identify specific patients at each contracted SPRS who were eligible for study participation. During the analytic phase of the study, service utilization and cost data available within claims were employed to assess healthcare utilization and calculate the cost of care for study participants in order to correlate patient activation with healthcare utilization.

### Managed Medicaid claims

Medical claims were provided for five states: Florida, Kansas, Missouri, New Hampshire, and Texas with 2 years of retrospective history from extraction date (October 1, 2015 to October 1, 2017).

### Survey

The survey battery included two validated surveys, the Patient Activation Measure–Mental Health (PAM-MH)^18^ and the Colorado Symptom Index (CSI).^19–21^ In addition, a number of items including demographics, socioeconomic status, treatment, and social support items to characterize the population and their standard mental health treatment practices included

- Age
- Gender
- Current employment status
- Living situation
- Marital status
- Level of professional healthcare support

The PAM-MH and CSI have both been previously used within SMI populations and deemed appropriate for this sample of patients with schizophrenia, BD, and MDD.^19,22,23^

The Patient Activation of Mental Health (PAM-MH) Survey is a 13-item questionnaire that can be used to assess a mental health patient’s level of activation in their own care. With scores ranging from 0 to 100, higher PAM-MH scores correspond to greater activation, while lower scores suggest less activation in their own care. Furthermore, a patient’s PAM-MH score and corresponding level (levels 1–4, higher levels correspond to higher scores) have been shown to inversely correlate with healthcare costs, with higher scores and more activation correlating with lower healthcare costs.^12^

The CSI is a validated 13-question survey that assesses the overall level of symptom severity in mental health patients. The CSI score ranges from 0 to 100 with an effective range to 80 and a score of 30 has been proposed as an appropriate clinical cut-off score, where a score less than or equal to 30 is categorized as normal, or having no mental health issues, while scores greater than 30 suggest increasing levels of mental health symptomology and/or severity.^19^

### Participant recruitment

All participants were invited to take part in the study by their mental health provider who was enlisted as an SPRS. SPRSs used the patient lists provided by Health Analytics to recruit patients who are eligible for study inclusion. They validated patient inclusion criteria prior to notifying patients of the study. SPRSs informed potential participants of the purpose, goals, and requirements of the study. Interested patients were provided with a participant survey packet that included a sign-up sheet, introductory letter, informed consent form, and a copy of the survey battery. Site staff was on hand to answer patients’ questions about informed consent. Health Analytics contact information was also made available to SPRSs and patients for questions regarding the study and the informed consent process. Patients who completed the informed consent were then asked to complete the survey.

### Survey administration

The survey was administered in hardcopy or electronically via the patient packet provided by the SPRS. The PAM-MH and CSI have both been administered using paper and pencil platforms within an SMI population.^19, 22^ To ensure the sample population understood the study, survey instructions and the informed consent form were written in simple language. SPRS staff were available to answer patient questions about the informed consent or survey; Health Analytics contact information was also available to both SPRSs and patients for questions. Patients were asked to complete the survey at the SPRS office and, if taken on paper, return the completed survey to a staff member during the same visit. The SPRS ensured each survey was complete and sent the completed surveys and informed consent forms to Health Analytics.

### Informed consent

An informed consent form that apprises patients of their rights in study participation was presented with the survey. There were two methods to complete the informed consent document, online or hardcopy. The online informed consent was available via the same link as the patient survey and was presented prior to the survey. Upon finishing the informed consent, the patient was prompted to proceed to the survey. Double-click verification was employed for the online informed consent. For hardcopy packets, the informed consent was returned to Health Analytics by the patient or SPRS in the self-addressed envelope provided. All required signature lines or fields on the informed consent were highlighted to draw the patient’s attention. Surveys that were returned without an associated signed informed consent were excluded from the study.

### Data analytic procedures

#### Calculation of healthcare costs

Medical claims, pharmacy claims, and hospital admissions tables were collated for the *N* = 136 survey patients and within that claims with service dates between October 1, 2016 and October 1, 2017. To calculate costs within the medical claims file, each summed claim was categorized as Inpatient, Emergency Room, Outpatient Hospital, Outpatient office, and Other based on associated location service codes. Total medical costs included emergency room costs, inpatient hospital, professional and pharmacy costs, outpatient hospital cost, outpatient office cost, and other costs. The Other Cost category included medical claims with unassigned cost categories based on their associated location of service code. Total pharmacy costs were summed up to the patient level using pharmacy claims data which were then merged to the medical claims file. Total healthcare cost was then calculated by adding total medical cost and total pharmacy cost. In addition, a subset analysis of psychiatric utilization and cost was conducted. Only claims with ICD-10 or ICD-9 diagnosis codes for Sz, BD, and MDD were considered.

### Descriptive statistics

Descriptive statistics, including demographics, were evaluated for the entire survey sample as well as for each of the disease-specific survey cohorts. Frequencies and percentages were presented for categorical variables including gender, employment status, marital status, living status, case manager interaction, PAM-MH level, and specific responses to PAM-MH and CSI questions. Means and SDs are presented for continuous variables including age, PAM-MH raw score, and CSI score as well as for variables related to cost and service utilization.

Correlation and regression analysis were used to estimate the association between PAM-MH score and healthcare costs. Zero-order correlation coefficients were calculated for the PAM-MH score and costs including pharmacy cost, medical cost, and total healthcare cost. Differences in correlation strength and direction were evaluated for subgroups including disease state (i.e., MDD, BD, Sz) and gender. Regression analysis was used to further examine these relationships. Healthcare costs were regressed onto covariates including age, gender, marital and employment status, and CSI score. The independent variable of interest was PAM-MH score. In addition, because there was a meaningful difference in the correlation analysis by gender, a PAM × gender interaction term was entered into the model.

SAS Enterprise 7.13 (SAS Institute, Cary, NC) was utilized for data transformations and merging of data from different tables, computing descriptive statistics and producing correlation coefficients with *p* values.

## Results

The study questionnaire was completed by *N* = 136 patients with an SMI primary diagnosis of Sz (*N* = 43), BD (*N* = 59), or MDD (*N* = 34). Table 1 presents the demographics for all study populations. Gender representation was nearly equal in the overall study group (female 57%), while major depression (71%) and BD (63%) had significantly more women as a part of their respective study cohorts. The average age among the bipolar (mean ± SD) (41.3 ± 15.2) and MDD (40.4 ± 14.5) patient cohorts was similar to the total sample (43.12 ± 14.3), while the Sz patient cohort was slightly older (47.7 ± 12.0). Over half (56%) of the total sample was unemployed. Most patients were single, never married. In the overall study population, many patients lived alone (36%) or with family (38%). Nearly half of Sz patients lived alone (47%), while 46% of bipolar patients lived with family. Most of the sample reported having no caregiver (74%), and very few lived with a roommate or in a group home. Interaction with a case manager was mixed between no interaction (43%) and weekly interaction (42%).

**Table 1:** Patient demographics

	Total		Schizophrenia		BD		MDD		*p* value
	*n* = 136		*n* = 43		*n* = 59		*n* = 34		
	Mean/f	SD/%	Mean/f	SD/%	Mean/f	SD/%	Mean/f	SD/%	
Age	43.1	14.3	47.7	12	41.3	15.2	40.4	14.5	**0.0352**
Gender									**0.0128**
Female	78	57%	17	40%	37	63%	24	71%	
Employment status									
Full time	10	7%	1	2%	5	8%	4	12%	0.7342
Part time	27	20%	7	16%	12	20%	8	24%	
Job training program	8	6%	3	7%	3	5%	2	6%	
Retired	15	11%	4	9%	6	10%	5	15%	
Unemployed	76	56%	28	65%	33	56%	15	44%	
Marital status									0.4363
Single	85	63%	31	72%	37	63%	17	50%	
Married	13	10%	2	5%	7	12%	4	12%	
Separated	7	5%	1	2%	3	5%	3	9%	
Divorced	26	19%	8	19%	11	19%	7	21%	
Widowed	5	4%	1	2%	1	2%	3	9%	
Living status									0.0672
Live alone	49	36%	20	47%	17	29%	12	35%	
Live with family	51	38%	14	33%	27	46%	10	29%	
Live with roommate	16	12%	1	2%	10	17%	5	15%	
Live with caregiver	0	0%	0	0%	0	0%	0	0%	
Group home or residential unit	20	15%	8	19%	5	8%	7	21%	
Caregiver									0.1509
Part-time caregiver	17	13%	6	14%	5	8%	6	18%	
Full-time caregiver	18	13%	4	9%	6	10%	8	24%	
No caregiver	101	74%	33	77%	48	81%	20	59%	
Case manager interaction									**0.0044**
Daily	3	2%	2	5%	0	0%	1	3%	
Weekly	57	42%	28	65%	23	39%	6	18%	
Monthly	8	6%	2	5%	3	5%	3	9%	
Every couple of months	9	7%	1	2%	5	8%	3	9%	
No case manager	59	43%	10	23%	28	47%	21	62%	

### Response to PAM-MH survey

**Figure 1:**
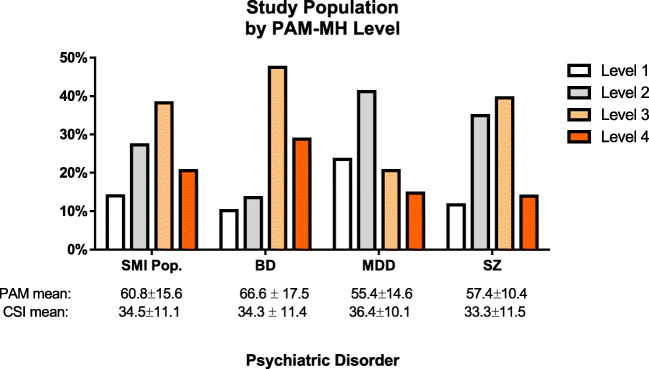
Frequency distribution (%) of the study population grouped by psychiatric disorder and PAM-MH level

The average PAM-MH score for the sample (*N* = 136) was 60.9 ± 15.6 indicating moderate patient activation (PAM-MH level 3; see Tables 2, 3 and Fig. 1). BD patients were the most highly activated (PAM-MH 66.6 ± 17.5), while MDD (55.4 ± 14.6) and Sz (57.4 ± 10.4) patients’ average activation scores were lower (*p* < 0.01) (Tables 2 and 3).

**Table 2:** Average PAM-MH and CSI total scores among each psychiatric disorder

	Total		Schizophrenia		BD		MDD		*p* value
	*n* = 136		*n* = 43		*n* = 59		*n* = 34		
	Mean/f	SD/%	Mean/f	SD/%	Mean/f	SD/%	Mean/f	SD/%	
PAM-MH total score	60.9	15.6	57.4	10.4	66.6	17.5	55.4	14.6	**0.0006**
CSI total score	34.5	11.1	33.3	11.5	34.3	11.4	36.4	10.1	0.4639

**Table 3:** Average PAM-MH and CSI total scores by gender

	Males		Females		*p* value
	*n* = 58		*n* = 78		
	Mean/f	SD/%	Mean/f	SD/%	
PAM-MH total score	59.2	17	62.1	14.5	0.28
CSI total score	33.3	11.4	35.4	11	0.28

### Response to Colorado Severity Index

The CSI measures the severity of psychological and emotional symptoms. The CSI score for the total sample (34.5 ± 11.1) indicated a moderate level of psychological symptomology. Although not statistically significantly different (Tables 2 and 3), the major depressive group (36.4 ± 10.1) was the most severe with BD (34.3 ± 11.4) and Sz (33.3 ± 11.5) groups slightly lower in overall symptom severity (Fig. 1, Tables 2 and 3).

### Healthcare service utilization and costs

Healthcare service utilization and costs are presented in Table 4 for the full survey population and each disease group. Office/clinic visits were the highest among the Sz group (reported per member per year (PMPY)) (45.0 ± 62.5), followed by the bipolar (35.6 ± 56.1) and the major depressive patient groups (25.0 ± 42.6). Outpatient hospital visits community mental health centers (CMHCs) were also highest among the Sz group and occurred at approximately three and two times the rate of bipolar and MDD patients, respectively. Emergency room (ER) utilization was nearly identical in the bipolar (3.1 ± 6.9) and MDD (2.9 ± 5.3) groups while the Sz group had the fewest ER visits (1.3 ± 2.6).

**Table 4:** Healthcare cost and service utilization

	Total		Schizophrenia		BD		MDD		
	*n* = 136		*n* = 43		*n* = 59		*n* = 34		
	Mean/f	SD/%	Mean/f	SD/%	Mean/f	SD/%	Mean/f	SD/%	*p* value
Service utilization									
Office/clinic visits	36.0	55.4	45.0	62.5	35.6	56.1	25.0	42.6	0.2926
Outpatient hospital visits	36.0	41.3	59.0	46.6	22.0	29.3	31.9	40.8	**0.0001**
ER visits	2.5	5.5	1.3	2.6	3.1	6.9	2.9	5.3	0.2275
Inpatient admissions	0.15	0.44	0.12	0.4	0.18	0.47	0.14	0.44	0.7238
Inpatient days	0.76	2.5	0.7	2.4	0.66	2.0	1.0	3.4	0.8089
% w/ ER visit	0.324	0.47	0.26	0.44	0.34	0.48	0.38	0.49	0.1899
% w/IP admission	0.03	0.17	0.02	0.15	0.03	0.19	0.03	0.17	0.6661
Rx fills	44.6	47.8	44.8	50.3	39.0	46.3	54.0	47.2	0.3463
Healthcare costs	*Mean*	*SD*	*Mean*	*SD*	*Mean*	*SD*	*Mean*	*SD*	
Office/clinic costs	$7358	13,521	$6819	12,682	$6109	12,246	$10,208	16,375	0.3556
Outpatient hospital costs	$9580	14,835	$12,320	13,360	$7466	16,101	$9781	14,132	0.2647
ER costs	$1135	3551	$1431	5013	$960	2740	$1063	2497	0.7989
Inpatient hospital costs	$899	3311	$1350	4953	$661	2236	$742	2106	0.5575
*Other healthcare costs	$1080	5256	$248	403	$529	1180	$3088	10,236	**0.0340**
Total medical costs	$20,057	23,190	$22,168	22,200	$15,724	22,772	$24,888	25,249	0.1500
Pharmacy costs	$4002	7771	$5452	10,031	$3248	6622	$3475	6154	0.3337
Total healthcare costs	$24,013	25,707	$27,620	24,725	$18,972	25,399	$28,358	26,641	0.1368

Generally, healthcare costs were highest among the Sz patient group for each service category relative to the other study groups (Table 4). Among the costs that were highest in the Sz group were the outpatient ($12,320 ± 13,360) and office/clinic ($6819 ± 12,682), which were then followed by pharmacy ($5452 ± 10,031), ER ($1431 ± 5013), and inpatient hospital ($1350 ± 4943) costs. Two notable exceptions of lower costs in the Sz cohort were relative to the major depressive cohort in the categories of office/clinic visits and other healthcare costs. Despite high costs among the Sz group in each service category, the major depressive group had the greatest overall total healthcare costs ($28,388 ± 25,249). These costs were primarily attributed to office/clinic ($10,208 ± 16,375), outpatient hospital costs ($9781 ± 14,132), and other healthcare costs ($3088 ± 10,236). Other healthcare costs included location services like non-residential substance abuse treatment facility, rural health clinic, and intermediate care facility. The BD patient cohort had the lowest total medical ($15,724 ± 22,772) and total healthcare ($18,972 ± 25,399) costs of all groups. In the BD group, outpatient hospital ($7466 ± 16,101) and office/clinic costs ($6109 ± 12,246) contributed the most to total medical costs, which were followed by ER ($960 ± 2740), inpatient hospital ($661 ± 2236), and finally Other healthcare costs ($529 ± 1180).

### Correlations between PAM-MH score and service utilization and costs

Analysis of total medical and healthcare costs by PAM-MH level in the BD group revealed that costs decrease significantly as the activation level increases (Table 5). Pearson zero-order correlation coefficients between PAM-MH score and service utilization revealed that PAM-MH scores were inversely related to total medical costs (*r* = − 0.207, *p* = 0.02) and total healthcare costs (*r* = − 0.194, *p* = 0.02) in the full sample (Table 6). These correlations were stronger for males (*r* = − 0.37, *p* = 0.005) for total medical costs and (*r* = − 0.34, *p* = 0.087) for total healthcare costs (Table 7). However, correlations for total medical and healthcare costs were weaker for females (Table 7). An inverse relationship between PAM-MH score and total medical cost approached statistical significance in the bipolar group (*r* = − 0.252, *p* = 0.054), but not in the MDD (*r* = 0.004, *p* = 0.99) or Sz (*r* = − 0.189, *p* = 0.25) groups (Table 6). Similarly, an inverse relationship between PAM-MH score and total healthcare costs for the bipolar group (*r* = − 0.232, *p* = 0.08) was observed that approached statistical significance. Finally, the bipolar group had a statistically significant positive correlation between PAM-MH score and total office visits (*r* = 0.258, *p* = 0.05), but negative correlations for total outpatient hospital (CMHC) visits (*r* = − 0.191, *p* = 0.15) and total prescription fills (*r* = − 0.191, *p* = 0.15).

**Table 5:** Total medical and healthcare costs by PAM-MH level in BD patients

	Level 1 (least activated)		Level 2		Level 3		Level 4 (most activated)	
	Mean	SD	Mean	SD	Mean	SD	Mean	SD
Total healthcare costs	28,503.28	33,650.94	23,152.83	29,159.97	18,372.9	27,806.77	12,335.23	12,764.46
Total medical costs	24,163.58	28,454.55	22,364.72	29,420.91	15,438.31	24,337.45	7798.1	5993.62

**Table 6:** Correlations between PAM-MH score and healthcare costs and service utilization

		PAM-MH total score—schizophrenia *N* = 43	PAM-MH total score—BD *N* = 59	PAM-MH total score—MDD *N* = 34	PAM-MH total score—total sample *N* = 136
Total office visits	Pearson correlation	0.021	0.258	− 0.049	0.127
	Sig. (2-tailed)	0.896	**0.049**	0.784	0.140
Total office costs	Pearson correlation	0.121	− 0.141	0.088	− 0.044
	Sig. (2-tailed)	0.440	0.287	0.622	0.608
Total OP hospital visits	Pearson correlation	0.052	− 0.191	0.185	−0.096
	Sig. (2-tailed)	0.741	0.148	0.296	0.266
Total OP hospital costs	Pearson correlation	− 0.456	− 0.244	0.022	− 0.245
	Sig. (2-tailed)	**0.002**	0.063	0.904	**0.004**
ER visits dichotomized	Pearson correlation	− 0.094	0.025	0.308	0.072
	Sig. (2-tailed)	0.548	0.853	0.077	0.403
Total ER costs	Pearson correlation	0.123	0.073	− 0.022	0.048
	Sig. (2-tailed)	0.430	0.585	0.901	0.583
Total admissions dichotomized	Pearson correlation	− 0.066	0.094	− 0.042	0.047
	Sig. (2-tailed)	0.675	0.480	0.813	0.590
Total hospital days	Pearson correlation	− 0.063	0.062	− 0.134	− 0.043
	Sig. (2-tailed)	0.690	0.641	0.451	0.622
Total medical costs	Pearson correlation	− 0.189	− 0.252	0.004	− 0.207
	Sig. (2-tailed)	0.224	0.054	0.981	**0.015**
Total fills	Pearson correlation	0.050	− 0.191	− 0.040	− 0.124
	Sig. (2-tailed)	0.749	0.148	0.820	0.151
Total Rx costs	Pearson correlation	0.175	− 0.035	− 0.145	− 0.017
	Sig. (2-tailed)	0.263	0.795	0.414	0.848
Total healthcare costs	Pearson correlation	− 0.095	− 0.232	− 0.030	− 0.194
	Sig. (2-tailed)	0.543	0.078	0.867	**0.024**

Correlation is significant at the 0.01 level (2-tailed)

**Table 7:** PAM-MH correlations to cost and service utilization

	Total medical	Total health
PAM-MH (all disorders, male, *N* = 58)		
*R* value	− 0.36669	− 0.34131
*p* value	**0.0046**	**0.0087**
PAM-MH (all disorders, female, *N* = 78)		
*R* value	− 0.00841	− 0.01327
*p* value	0.9417	0.9082

In general, MDD and Sz groups showed weak, non-significant correlations with total healthcare costs; however, there was a strong inverse relationship that occurred between total outpatient hospital cost (*r* = − 0.456, *p* = 0.002) and PAM-MH score among Sz patients. Correlations to cost and service utilization in the MDD population were similar to that observed in the Sz population in that most, with the exception of ER visits, were weak and not statistically significant.

#### Regression modeling of total medical costs

**Figure 2:**
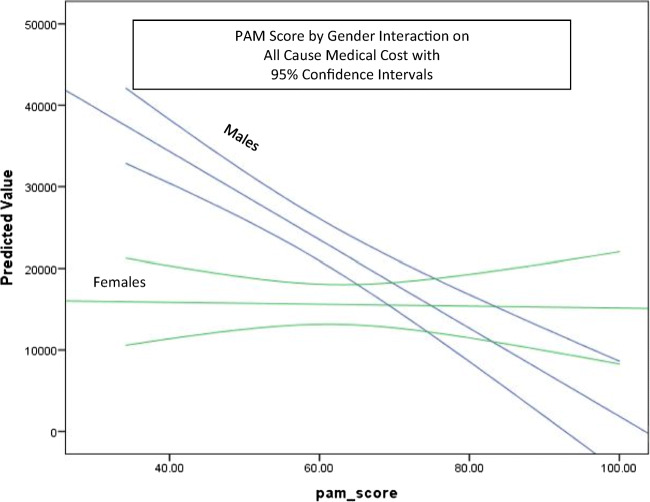
PAM score by gender interaction on all-cause medical cost with 95% confidence intervals

Because correlational analysis showed an inverse and statistically significant relationship between PAM-MH score and total medical costs, regression modeling was performed to control for the effect of covariates on the relationship between cost of care and activation. Independent variables in the regression model included age, gender, employment status (employed vs. unemployed), marital status (married vs. not), disease state, disease severity (CSI), and the predictor of interest, PAM-MH score (Tables 8 and 9). In addition, because the correlational analysis indicated a difference in the relationship between PAM-MH and cost by gender, a PAM × gender interaction term was entered into the models. Dependent variables included pharmacy cost, medical cost, and total cost of care. The regression models predicted medical cost (*R*^2^ = 0.309, *p* < 0.01) (Table 9) and total cost of care (*R*^2^ = 0.234, *p* < 0.05) (Table 8) but failed to predict pharmacy cost (*R*^2^ = 0.03, NS). The main effect of PAM-MH score contributed significantly to both the medical cost model (PAM-MH score *b* = −1033.65, *t* = −2.31, *p* < 0.05)^1^ and the total cost of care model (PAM-MH score *b* = − 311.64, *t* = − 2.31, *p* < 0.05). In the medical cost model, age, gender, employment, and marital status all contributed to the model (*p*s < 0.05) as did the main effect for PAM-MH score. However, the PAM × gender interaction term (*b* = 467.51, *t* = 2.091, *p* < 0.05) contributed significantly indicating that the relationship between PAM-MH and medical cost is conditional on gender (Fig. 2). The total cost of care model returned similar results with fewer covariates contributing and the PAM × gender interaction term showing a non-significant trend (*p* = 0.081) in the same direction as was seen in the medical cost model. In the medical cost model, a single point increase in PAM-MH score is associated with a $467.51 greater decrease in total cost PMPY for males compared with females with all other measures being held constant.

**Table 8:** Regression model all-cause medical cost = age + gender + employment status + marital status + dx + CSI score + PAM score + PAM score × gender

Variable	Estimate	Standard error	*p* value	95% CI	
Intercept	131,446.25	23,384.55	< 0.001	69,932.39	128,802.68
Age	286.52	133.89	0.034	21.56	551.48
Gender	− 32,078.71	14,181.27	0.025	− 60,143.03	− 4014.39
Employment status	− 18,045.36	3946.21	< 0.001	− 25,854.79	− 10,235.92
Marital status	− 16,517.45	5791.90	0.005	− 27,979.45	− 5055.44
Diagnosis MDD	5760.09	4750.25	0.228	− 3640.51	15,160.69
Diagnosis BD	1560.83	4348.64	0.72	− 7044.99	10,166.65
CSI score	− 297.96	168.30	0.079	− 631.02	35.10
PAM score	− 1033.65	358.04	0.005	− 895.92	− 236.35
PAM × gender interaction	467.51	223.59	0.039	25.04	909.99
***R***^**2**^		***F***	**Significance**	**Total medical cost mean**	
0.309		6.249	< 0.0001	20,057

**Table 9:** Total cost of care = age + gender + employment status + marital status + dx + CSI score + PAM score

Variable	Estimate	Standard error	*p* value	95% CI	
Intercept	96,009.66	16,283.05	< 0.001	61,924.64	126,019.84
Age	237.30	153.86	0.125	− 67.16	541.76
Gender	− 2037.42	4164.37	0.626	− 10,277.96	6203.11
Employment status	− 18,384.18	4550.12	< 0.001	− 27,388.04	− 9380.33
Marital status	− 17,737.02	6672.12	0.009	− 30,939.94	− 4534.09
Diagnosis MDD	2408.76	5473.67	0.661	− 8422.65	13,240.16
Diagnosis BD	− 3062.78	4930.15	0.536	− 12,818.66	6693.11
CSI score	− 292.18	192.53	0.132	− 673.16	88.81
PAM score	− 311.64	141.60	0.03	− 591.84	− 31.43
***R***^**2**^		***F***	**Significance**	**Total medical cost mean**	
0.234		4.838	< 0.001	24,013

## Discussion

Quality measures and lower total healthcare costs have become a major focus of US healthcare reform. Undoubtedly, this trend toward adopting new quality measures will continue into the foreseeable future, as pressures to contain medical costs in an aging population intensify. Indeed, within the last decade, the Centers for Medicare and Medicaid Services (CMS) implemented several value-based programs, with the goal of linking provider payment to performance and quality measures. Furthermore, CMS recently announced an impending roll-out of five value-based primary care models scheduled to begin in 2020. This rise in accountability and financial risk has re-directed providers and payors toward understanding patient’s contribution to treatment fidelity, health outcomes, and cost. This interest is likely to place patient engagement (like the PAM, PAM-MH), and new digital technologies that measure drivers of treatment outcomes and cost such as adherence can potentially add greater definition of the drivers of medication value and influence value-based reimbursement.

To our knowledge, this is the first study estimating the association between patient activation, or engagement, and healthcare service utilization and costs within an SMI population using the PAM-MH. In the current study, 136 mental health patients completed a survey battery including the PAM-MH and CSI, and responses were merged with administrative claims data at the claimant level allowing for analysis of patient-reported outcomes, service utilization, and cost of care during a 1-year period. PAM-MH score was found to be inversely related to total outpatient hospital costs, total medical costs, and total healthcare costs in the full SMI study sample (Table 6). These correlations were magnified by gender differences, with males showing a more robust, inverse correlation for total medical and healthcare costs when compared with females. Overall, these results are similar to what has been previously described in other disease states like cancer and diabetes where increased activation correlates with lower healthcare costs.^24,25^

Of the three groups analyzed, BD patients were found to be significantly more activated compared with the Sz group while the MDD group was the least activated. While the full SMI group showed statistically significant correlations in some health measures, correlations between PAM-MH score and bipolar diagnosis were just below the level of statistical significance for total outpatient hospital costs, total medical costs, and total healthcare costs. Interestingly, the Sz group had the highest inverse correlation for total outpatient hospital costs and PAM-MH score, suggesting that increases in PAM-MH score can drastically reduce care-related cost among this population.

Results from the correlational analysis were further explored using regression analysis. The significant interaction between PAM-MH score and gender on cost of care suggests that activation level influences male more than female utilization of high-cost healthcare services. Based on bivariate results, there is no support for this effect being an artifact of differences between genders on mean PAM-MH score. As males become more activated, they become more active in managing their own healthcare. By contrast, within this sample, females remain moderately involved in managing their healthcare regardless of PAM-MH score. The implications of this finding, were it to be replicated and verified, could include developing different intervention targeting algorithms for men and women, particularly when raising the level of activation is the intent of the intervention. Similar to Lindsay et al.,^26^ the current results suggest that a single point increase in PAM-MH score could result in significant annual cost avoidance ($1033 per male and $566 per female member per year). Similarly, decreases in PAM-MH score are associated with increases in total medical and total healthcare costs. While there are undoubtedly many factors that play into a patient’s healthcare service utilization and medical costs, level of activation seems to be of some incremental importance. It is clear that information on their patient’s level of activation could be of importance to SMI providers as they are developing and managing care plans. However, currently unclear are the circumstances under which an SMI patient’s level of activation can be influenced and the range of level of response that can be expected in response to specific interventions. Would programs and new digital technologies designed to enhance patient activation influence it enough to change the course of treatment outcomes? Future studies employing a longitudinal prospective design in a larger SMI population may be of significance for answering these and other questions.

### Study limitations

The present study was limited by its sample size (*N* = 136), its limited geographical coverage (state of Kansas), and its restriction to a national managed Medicaid organization patient population. While it is possible that these results will extend to other populations, studies should be conducted in broader populations including patient populations with commercial insurance. Further, as the present study was cross-sectional in design, the results are suggestive but not causal. Longitudinal studies that include interventions that influence the level of patient activation are needed.

### Implications for behavioral health

Good outcomes among the treated SMI populations are often driven by a combination of medication, psycho-social support, and healthy behaviors to name a few. The relationship between patient activation and good outcomes in general medicine is well documented. In the SMI treatment literature, the relationship between patient engagement in healthy behaviors, good medication adherence, participation with psycho-social interventions, and positive outcomes is also well documented. The present paper provides some support for increasing patient activation among SMI patient populations in the service of improving outcomes both of which are valuable to patients, their family members, clinicians, care managers, and other mental health stakeholders.

## Footnotes

1 This main effect is not reported in Table 8 as it derives from the main effects model.
